# The Role of Autophagy-Related Proteins in *Candida albicans* Infections

**DOI:** 10.3390/pathogens5020034

**Published:** 2016-03-29

**Authors:** Jenny M. Tam, Michael K. Mansour, Mridu Acharya, Anna Sokolovska, Allison K. Timmons, Adam Lacy-Hulbert, Jatin M. Vyas

**Affiliations:** 1 Department of Medicine, Division of Infectious Diseases, Massachusetts General Hospital, Harvard Medical School, Boston, MA 02114, USA; jmtam@mgh.harvard.edu (J.M.T.); mkmansour@mgh.harvard.edu (M.K.M.); anyasokol@gmail.com (A.S.); atimmons1@mgh.harvard.edu (A.K.T.); 2 Immunology Program, Benaroya Research Institute, Seattle, WA 98101, USA; macharya@benaroyaresearch.org (M.A.); adamlh@benaroyaresearch.org (A.L.-H.)

**Keywords:** autophagy, LC3-associated phagocytosis (LAP), *Candida albicans*, *Aspergillus fumigatus*, LC3, innate immunity, Dectin-1

## Abstract

Autophagy plays an important role in maintaining cell homeostasis by providing nutrients during periods of starvation and removing damaged organelles from the cytoplasm. A marker in the autophagic process is the reversible conjugation of LC3, a membrane scaffolding protein, to double membrane autophagosomes. Recently, a role for LC3 in the elimination of pathogenic bacteria and fungi, including *Candida albicans* (*C. albicans*), was demonstrated, but these organisms reside in single membrane phagosomes. This process is distinct from autophagy and is termed LC3-associated phagocytosis (LAP). This review will detail the hallmarks of LAP that distinguish it from classical autophagy and review the role of autophagy proteins in host response to *C. albicans* and other pathogenic fungi.

## 1. Introduction

Invasive candidiasis, an infection caused by the ascomycete fungus *Candida albicans* (*C. albicans*), is a leading cause of death among immunocompromised patients over the last 20 years [1]. Candidemia has a crude mortality rate exceeding 50% [2] and is the fourth most common bloodstream infection in hospitalized settings in the US and Europe [1]. In order to protect the host from fungal pathogens like *C. albicans*, the immune system relies on innate immune phagocytes including antigen presenting cells (APCs) as the first line of defense to seek, recognize, and destroy foreign pathogens. Phagocytes and APCs such as macrophages, neutrophils, and dendritic cells (DCs) engulf and neutralize pathogens through a process called phagocytosis. Engulfed pathogens are processed and degraded in organelles termed phagosomes that form around the internalized material. Phagocytes use pattern recognition receptors (PRRs), such as Toll-like receptors (TLRs) and C-type lectin receptors (CLRs), like Dectin-1, to recognize fungi. TLR and CLR signaling triggers the phagocytic machinery and production of inflammatory cytokines and intercellular signaling molecules, which direct an immune response to the site of infection [3,4]. Each type of PRR recognizes conserved molecular motifs called pathogen associated molecular patterns (PAMPs) that distinguish the pathogen from the host.

Recently, autophagy has been linked to host defense against bacterial and fungal organisms [5]. Autophagy is a physiological cellular process whereby intracellular components undergo lysosomal degradation and recycling. Historically, autophagy has been recognized as playing an important role in maintaining cell homeostasis and cell metabolism; it provides nutrients during periods of starvation and removes aged or damaged organelles from the cytoplasm. However, an increasing number of reports demonstrate that autophagic proteins have a significant role in defense against fungal pathogens including *C. albicans* [6,7,8,9,10], *Aspergillus fumigatus* (*A. fumigatus)* [11,12,13,14], and *Cryptococcus neoformans* (*C. neoformans)* [15]. Autophagy proteins, such as microtubule-associated proteins 1A/1B light chains 3A/LC3A and 3B/LC3B (MAP1LC3A/B or LC3), are recruited to the pathogen-containing phagosome following phagocytosis [16,17]. In phagocytosis and autophagy, the recruitment of specific proteins to the (auto)phagosomal membrane is a critical event that leads to degradation of its contents. The recruitment of autophagy proteins to the phagosomal membrane represents a distinct process in host defense and is an active area of research. This review will focus on the mechanisms of fungal recognition and phagocytosis, and explore how autophagy proteins are recruited to the phagosomal membrane and affect immunity towards *C. albicans* and other pathogenic fungi.

## 2. Fungal Recognition by Dectin-1

Clearance of fungal pathogens like *C. albicans* begins with recognition of the organism by PRRs such as Dectin-1, a type II membrane protein and CLR highly expressed on phagocytes [18]. Dectin-1 recognizes the carbohydrate epitope β-1,3-glucan [19], which constitutes the major cell wall component of pathogenic fungi including *C. albicans* [20]. Dectin-1 is required for proper modulation of immune responses. Patients with mutations in Dectin-1 are at higher risk for invasive fungal infections [21,22], and autoimmune colitis driven by *C. albicans* [23]. The cytoplasmic tail of Dectin-1 contains an immunoreceptor tyrosine-based activation (ITAM)-like motif [24], similar to T cell receptors, B cell receptors, and Fc receptors. While a typical ITAM contains two tyrosines that are used for signaling, the motif in Dectin-1 has a single tyrosine residue and is thus referred as a “hemITAM” [25,26]. Upon pathogen recognition by the extracellular domain of Dectin-1, the tyrosine residue within the cytoplasmic hemITAM is phosphorylated [25,27] by Src family kinases, which then recruit and activate spleen tyrosine kinase (Syk) [28]. These kinases trigger recruitment and activation of nicotinamide adenine dinucleotide phosphate (NADPH) oxidase [29], which leads to release of antimicrobial reactive oxygen species (ROS) into the phagosome. Further Dectin-1 activation triggers inflammatory responses, including production of the cytokines, TNF-α and IL-12 [30], through activation of nuclear factor of activated T cells (NFAT) and nuclear factor kappa-light-chain-enhancer of activated B cells (NF-κB) as well as phagosome maturation, a process of acidification and lysosomal fusion [31]. Several transmembrane TLRs including TLR1, TLR2, TLR4, and TLR6 coordinate with Dectin-1 for fungal recognition [32]. Interestingly, TLR9, which is located on intracellular membranes, has also been implicated in antifungal defense involving *C. albicans* [33]. Although the best-known PAMP for TLR9 is unmethylated bacterial and viral CpG-rich DNA, TLR9 has been shown to be present on phagosomes containing *C. albicans* [34,35]. Recognition of β-1,3-glucan by Dectin-1 triggers localization of TLR9 to phagosomes. Furthermore, TLR9-dependent changes in gene expression are specifically regulated by Dectin-1 [36].

## 3. Autophagy

**Figure 1:**
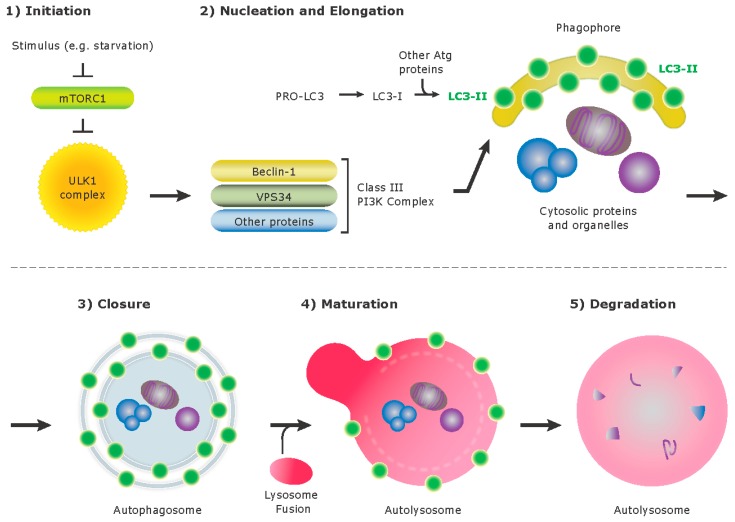
Simplified model of canonical autophagy that leads to degradation of cellular components.

Autophagy is an intracellular degradation system that delivers cytoplasmic materials to lysosomes (Figure 1). There are three different types of autophagy: macroautophagy, chaperone mediated autophagy, and microautophagy. In mammalian cells, macroautophagy, herein referred to as “autophagy”, is characterized by the formation of double membrane vesicles termed autophagosomes, which sequester damaged organelles, protein aggregates, or invading pathogens for degradation. Constitutive autophagy is required for cellular housekeeping such as removing damaged organelles and as a protective mechanism against cellular stress. The autophagy pathway can be initiated by various stimuli, such as starvation, and these triggers are transduced through the suppression of mammalian target of rapamycin (mTOR), which induces the formation of an isolation membrane or “phagophore” and involves multiple components including ULK1 (Unc-51-like kinase1). This process leads to the recruitment of the class III phosphatidylinositol-3-OH kinase (PI(3)K) complex, which includes VPS34, Beclin-1, and promotes the formation of the autophagosome. A complex of the Atg proteins, Atg12-Atg5-Atg16L1, is present on the outer membrane and the microtubule associated protein1 light chain 3 (MAP1 LC3 known as LC3-II) is present on both inner and outer membrane of the isolation membrane. The lipidated form of LC3, termed LC3-II, is conjugated to phosphatidylethanolamine, and is the most commonly monitored autophagy related protein.

Once cellular contents are enclosed within an autophagosome, the outer membrane of the autophagosome may fuse with endosomes and ultimately lysosomes, forming the autolysosomes that degrade the autophagolysosomal contents.

There is a growing appreciation for the complex role of autophagy proteins in immunity and inflammation. Autophagy plays a direct role in host defense by promoting the elimination of pathogens and indirectly by tight regulation of the innate and adaptive immune signaling pathways [37,38,39]. Engagement of various families of innate immune PRRs such as membrane-bound TLRs or cytosolic NOD-like receptors (NLRs) by pathogen ligands leads to the induction of autophagy. Detecting LPS from Gram-negative bacteria by TLR4 in macrophages drives TRAF-6-mediated ubiquitination of Beclin-1 [40]. Other TLR family members (TLR3, TLR5, TLR6, and TLR7) also induce autophagy through interaction of MyD88 and/or TRIF adaptor proteins with Beclin-1 [41,42]. Intracellular NLRs for bacterial peptidoglycan fragments Nod1 and Nod2 recruit the autophagy protein Atg16L1 to the plasma membrane at the bacterial entry site and promote wrapping of invading bacteria by autophagosomes [43]. In addition to PRRs, several pro-inflammatory cytokines including TNF-α, IL-1β, and both type I and type II interferons can induce autophagy and control infection [44,45,46]. On the other hand, autophagy negatively regulates activation of the inflammasome, which is formed after stimulation of NLR family receptors, and limits sequential IL-1β release through removal of damaged mitochondria and aggregated inflammasome structures. Therefore, the role of autophagy proteins in immunity and inflammation is complex and influences multiple pathways.

Autophagy and autophagy proteins are an important defense mechanism against many intracellular pathogens [47]. However, Atg-dependent defense mechanisms do not always involve autophagosome formation. Stimulation of autophagy by IFN-γ or rapamycin causes recruitment of Beclin-1 and LC3-II to *Mycobacterium tuberculosis*-containing phagosomes, which leads to the delivery of mycobacteria to mature double-membrane autophagolysosomes. Thus autophagy suppresses intracellular survival of mycobacteria in macrophages [48] and suppresses bacterial burden *in vivo* [49]. Sensing *Shigella flexneri* by Nod proteins also induces canonical autophagy and delivery of invading bacteria to autophagosomes [43]. IFN-γ/LPS-induced autophagy protein Atg5 is essential for *in vivo* resistance to *Listeria monocytogenes* and *Toxoplasma gondii*. Through the autophagosome-independent mechanism Atg5 recruits Irga6 GTPase to bacteria-containing or parasitophorous vacuole membrane and induces IFN-γ-mediated clearance of pathogens [50].

During viral infections, triggers such as cell stress (ER stress [51] and ROS [52]), viral ligands for PRRs [41,53], and cytokines [54] can induce autophagy in the cell. Canonical autophagy controls viral replication directly through removal of viral particles, proteins or replication complexes by degradation in lysosomes [55,56]. Additionally, autophagy machinery components have also been shown to play a role in the delivery of viral components to TLR-signaling or major histocompatibility complex (MHC)-loading compartments, which induce IFN type I [57,58] production and MHC class I and MHC class II antigen presentation [59,60,61].

Overall, autophagy is a dynamic process that operates at a basal level to promote general cellular homeostasis and it can also be induced in response to cellular stress. Autophagy proteins can contribute to many cellular processes outside of canonical autophagy signaling and are an active area of research. Further investigation is necessary to uncover the complex and influential role of autophagy and autophagy proteins in cell biology.

## 4. LC3-Associated Phagocytosis (LAP)

More recently, it has become apparent that autophagy proteins such as LC3 and Atg5 are involved in broader processes of membrane trafficking and organizational events in cells. These proteins have now been linked to internalization of microbial and self-particles in a process termed LC3 associated phagocytosis (LAP). This process involves single membrane phagosomes that directly associate with LC3, ultimately leading to the degradation of the phagosomal contents. Several features distinguish LAP from autophagy: LAP lacks the double membrane autophagosome and autophagy requires activity of a pre-initiation complex (including ULK1) but LAP does not. However, LAP requires the components of autophagy pathway such as Beclin1, Atg5 and Atg7. Class III PI3K associated protein Rubicon (RUN domain protein as Beclin-1 interacting and cysteine-rich containing) has also been shown to be important for LAP [13].

**Figure 2:**
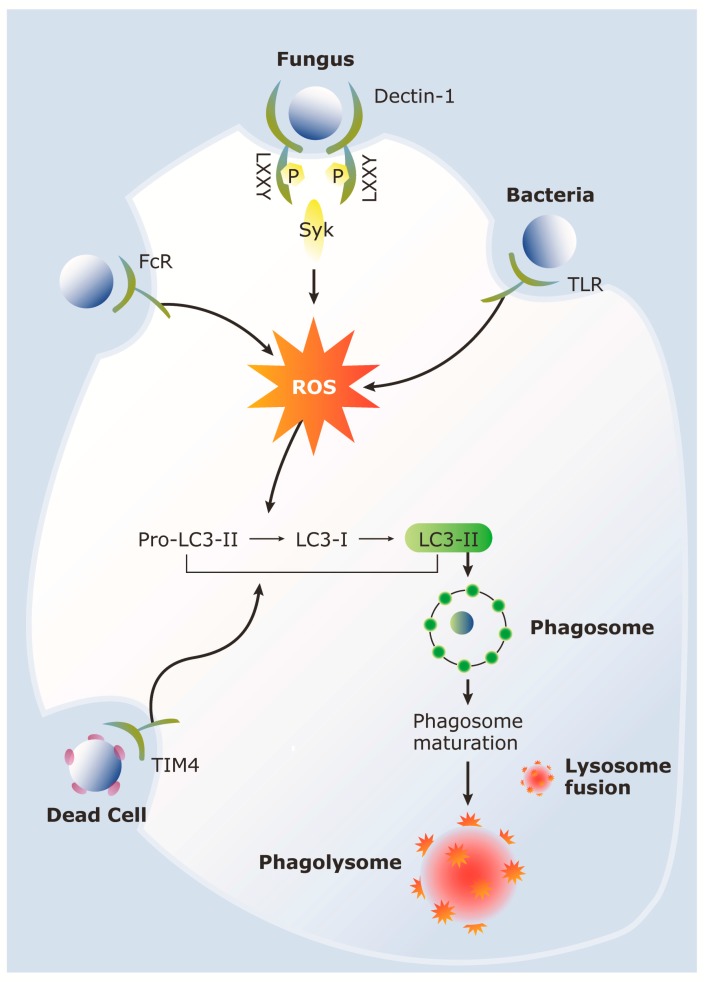
Cell surface receptors recognize a variety of particles inducing LAP. Initiation of LAP leads to ROS generation and subsequent LC3 processing and recruitment of LC3-II to the phagosomal membrane. The phagosome then matures and fuses with the lysosome to form the phagolysosome for particle degradation.

LAP occurs in response to engagement of particles that stimulate cell surface receptors such as TLRs and Fc receptors [62] (Figure 2). LAP is also apparent upon engulfment of dying cells by TIM4 [13], engulfment of living cells by mammary epithelial cells [63], and through fungal antigen recognition by Dectin-1 (Figure 2). Functionally, LAP has been implicated in accelerated phagosome maturation in the context of particles containing TLR ligands [64], for optimal degradation of dead cell cargo in macrophages [65] and degradation of photoreceptor outer segments by retinal pigment epithelial cells [66]. Therefore, LAP is a widespread mechanism for processing and degradation of cargo in phagocytic cells.

## 5. Role of LAP during Host Response to *C. albicans*

More details describing the mechanisms of fungal-initiated LAP has emerged from studies indicating roles for Syk kinase through the Dectin-1 signaling pathway (see Table 1 for summary of studies). Studies by Ma *et al.* showed that Dectin-1 triggered by fungal antigens in DCs led to recruitment of LC3 to *C. albicans* containing phagosomes. Although this pathway showed no difference in TNF-α and IL-6 production or *C. albicans* killing, it did facilitate MHC class II presentation of fungal antigens. This Dectin-1 triggered LC3 recruitment required Syk phosphorylation and ROS production by NADPH oxidase. In macrophages, however, our group showed a difference between LC3β**^−^**^/**−**^ and wild type primary macrophages in TNF-α, IL-6, and IL-1β production and *C. albicans* survival [10]. In contrast to studies in DCs, recruitment of LC3 to fungal phagosomes is important for fungicidal activity and expression of pro-inflammatory cytokines in macrophages. The distinct consequences of LC3 recruitment between DCs and macrophages could be due to the differences in phagosome maturation between the two cell types. NOX2 activity in macrophages generates a respiratory burst for effective microbicidal activity, and in DCs, ROS controls the redox potential and pH. As a result, relevant antigens are not rapidly degraded and can be loaded onto class I MHC molecules seen in cross-presentation and class II MHC molecules in the endolysosomal compartment.

These studies also link ROS production by phagosomal NADPH oxidase as a key signal for LC3 recruitment downstream of Syk activation. The role of ROS generation in leading to LC3 recruitment is also supported by other studies looking at antibacterial autophagy [67]. There is precedent for ROS signaling in starvation-induced autophagy and in this context the LC3 lipidation factor Atg4 is regulated by ROS [68] thus linking ROS generation to LC3 lipidation. More recent work by Ma *et al.* has also identified other autophagy related proteins such as FYCO1 that is recruited via this process to the fungal phagosome [69]. These studies allow us to put together a model in which Syk activation by cell surface receptors such as Dectin-1 could lead to ROS generation and recruitment of autophagy proteins to the phagosomes. These findings are important for our understanding of how the two ancient mechanisms of autophagy and phagocytosis intersect for efficient processing and clearance of bacterial and fungal pathogens, including *C. albicans*, and self derived particles.

The role of autophagy proteins in *in vivo C. albicans* infection models has been considered controversial. Autophagy proteins have shown to play protective role in host defense as seen in an *in vivo* murine *C. albicans* infection model [6,15] while some reports have shown that autophagy proteins are not critical for human host protection in *C. albicans* infection [8,9]. Nicola *et al.* showed that knockdown of Atg5 in J774.16 macrophages decreased LC3 recruitment to *C. albicans* containing phagosomes, and mice conditionally lacking Atg5 in myeloid cells, which include macrophages, DCs, and neutrophils, showed increased susceptibility to *C. albicans*. In contrast, upon stimulation with *C. neoformans*, the same J774.16 macrophages showed little LC3 recruitment, and there was no increased susceptibility to *C. neoformans* in mice conditionally lacking Atg5. Although Smeekens *et al.* show no difference in susceptibility to *C. albicans* in mice conditionally lacking autophagy-related protein 7 (Atg7), this group used a different strain of *C. albicans* and a conditional knockout mouse for a different autophagy protein. Nonetheless, these findings suggest that the role of autophagy proteins in host protection against fungal pathogens depends on the fungal species and may rely on specific autophagy components.

Another report also confirms the involvement of autophagy in murine host protection against *C. albicans* and sheds more light on the mechanism by which macrophages are involved in this process. Kanayama *et al.* used *C. albicans* to infect mice conditionally lacking Atg7 in myeloid cells, and these mice had decreased survival and increased fungal load in the spleen and kidney compared to their wild type counterparts [6]. Interestingly, a significant difference in weight reduction was seen 1–2 days post infection, which implicated the role of Atg7 in myeloid cells in host protection in early stages of fungal infection. The authors observed that autophagy was induced by *C. albicans* in macrophages, and autophagy proteins enhanced production of neutrophil chemoattractants *in vivo*, resulting in increased neutrophil recruitment. In addition, Atg7-deficient macrophages showed decreased NF-κB activity compared to wild type. NF-κB induces gene expression that promotes chemokine production, and Kanayama *et al.* further show that autophagy regulates chemokine expression by sequestering the NF-κB inhibitor, A20. Indeed, Atg-7 deficient macrophages had higher levels of A20 protein expression compared to wild type macrophages. The authors also show an association of A20 to p62 using confocal microscopy and immunofluorescence. p62 is an adaptor protein that sequesters and delivers proteins to the autophagosome, and p62 deficient macrophages showed increased levels of A20, which resulted in reduced chemokine production. In total, these results suggest a model by which *C. albicans* induces autophagy in macrophages upon infection, whereby p62 sequesters A20 in the autophagosome, induces NF-κB, and subsequently secretes chemokines to recruit neutrophils for fungal clearance.

Although autophagy is necessary for host protection in murine *C. albicans* infection models, different effects may be seen in humans. Smeekens *et al.* observe strong recruitment of LC3-II to *C. albicans*-containing phagosomes in HeLa cells [9]. However, following stimulation of *C. albicans* to human peripheral blood mononuclear cells, they did not see any effect on phagocytosis, cytokine production (TNF-α, IL-6, and IL-1β), or ability to kill the organisms upon autophagy inhibition in these cells. Other reports [8,9] further assessed the influence of autophagy in anti-*Candida* host defense by examining whether polymorphisms in autophagy genes correlated to susceptibility to *Candida* infections. Cells with genetic variations in ATG16L1 showed a significant difference in *C. albicans*-induced TNF-α production [8], but no differences in the production of 6 other cytokines. Another study examined 18 SNPs (single nucleotide polymorphism) in 13 different autophagy genes, and none were significantly associated with susceptibility to disseminated candidiasis. Furthermore, none of the 18 SNPs studied correlated with circulating cytokine concentrations in patients with candidemia or clinical outcome of disease. Results from this single study suggest that autophagy may be redundant for human host response against systemic *C. albicans* infections, but further investigations are necessary.

## 6. Role of Autophagy Proteins in *Aspergillus* Infections

In addition to pathogenic yeast, autophagy is involved in the control of invasive mold species (Table 1). Recent data suggests that autophagy plays a central role in the control of the most common mold infection, *Aspergillus fumigatus* (*A. fumigatus*). Patients at high risk for *A. fumigatus* infection include individuals with acquired immune deficiencies including the use of long-term corticosteroids, as well as individuals being treated for specific myeloid malignancies, such as acute myelogenous leukemia, where chemotherapeutics have rendered patients neutropenic or the malignancy itself is a functionally neutropenic state [70,71]. Finally, we also encounter rare patients with congenital immune mutations that result in increased susceptibility to invasive mold infection such as defects in NADPH oxidase complex manifesting clinically as chronic granulomatous disease (CGD) with marked impairment in neutrophil oxidative burst [72,73].

In a recent study by Kyrmizi *et al.* [11], the relationship between autophagy and *A. fumigatus* is explored. The pathogenesis of many mold infections, unlike yeasts, is through sinuopulmonary exposure of conidia or spores. Using co-cultures of human monocytes and *A. fumigatus* conidia, Kyrmizi *et al.* demonstrate that conidia are avidly phagocytosed with LC3 recruitment shown 2 h later by microscopy. Biochemical analysis for active, lipidated LC3 (LC3-II) confirms only live *A. fumigatus* conidia, but not killed spores, are capable of eliciting LC3-II formation peaking at 2–4 h following phagocytosis [11]. The cell wall of *Aspergillus* conidia is composed of a hydrophobic protein, rodA. Following phagocytosis, conidia have been observed to “swell” changing the cell wall ultrastructure to a composition more heavily decorated with carbohydrates including galactomannan and β-glucans. The authors show that indeed only conidia pre-treated to induce swelling are capable of activating LC3-II. Moreover, blocking monocyte recognition of β-glucans with competitive inhibitors or enzymatic digestion of β-glucans resulted in lower LC3-II suggesting a link between receptor-mediated recognition of β-glucans as a critical step in the activation of the autophagy pathway.

Dectin-1 is the dominant receptor used by the innate immune system for recognition of β-1,3- glucan. Using monocytes from patients with known Dectin-1 deficiencies, Kyrmizi *et al.* confirm that Dectin-1 recognition of *A. fumigatus* conidia is required for LC3-II formation but not TLR 2 or TLR4. The authors use chemical blockade of Src and Syk kinases, both essential for Dectin-1-mediated cell signaling to investigate the potential role of Dectin-1 between *Aspergillus* conidia and autophagy. Following chemical blockade of Syk and Src in wild-type monocytes incubated with swollen conidia, LC3-II formation was significantly abrogated [11]. The generation of ROS in response to *Candida* regulates autophagy through a Dectin-1-Syk dependent mechanism [10]. CGD patients harbor one of several mutations in the NADPH oxidase complex resulting in diminished ROS production and increased susceptibility to invasive fungal infections. Using primary monocytes from CGD patients, the authors determine that CGD monocytes co-cultured with *A. fumigatus* conidia fail to elicit or recruit LC3-II to swollen conidia. Furthermore, the authors silence transcription of Atg5 a key autophagy component, as well as inhibit ROS production using corticosteroids, both of which result in abrogation of LC3-II recruitment. Collectively, these data support activation of autophagy within monocytes in response to *A. fumigatus* conidia through a Dectin-1-Syk-ROS dependent mechanism.

LAP is involved in the elimination of molds including *A. fumigatus*. PRRs are the phagocytic trigger for LAP similarly to traditional phagocytosis. Recent work by Martinez *et al.* [13] demonstrates an essential role for Rubicon in LAP of *A. fumigatus*. Rubicon associates with the class III PI3K complex and negatively regulates autophagosome and endosome formation [13,74]. Using CRISPR-Cas9 to delete Rubicon had no affect on phagocytosis rates compared to wild-type, although LC3 is no longer recruited to ingested particles. Rubicon**^−^**^/**−**^ animals crossed to LC3-GFP mouse show an increased number of LC3 puncta suggesting that Rubicon can cross-regulate autophagy. To further define the influence of Rubicon on recruited host proteins, proteomic analysis was performed on purified LAP phagosomes, which revealed decreased Beclin-1, Atg7, UVRAG, VPS34 and LC3-II [13]. Moreover, the recruitment of Rubicon to LAP phagosomes was found to be dependent on PI(3)K complex; interruption of VPS34, a critical stability component for the PI(3)K complex shows absence of Rubicon recruitment to LAP phagosomes. Phagosome maturation of LAP phagosomes requires ROS through NOX2 (gp91PHOX). Martinez *et al.* find that ROS is required for successful LAP through the use of NOX2^−/−^ cells or chemical inhibition of ROS generation. In addition, phagosome maturation, the process of lysosomal fusion, was found to be dependent on active LC3-II through an unidentified mechanism [13]. These data define an essential role for Rubicon in LAP.

Martinez *et al.* extend their observations to demonstrate that control of *A. fumigatus* requires functional LAP. Macrophages deficient in Rubicon, Beclin-1 and NOX2 or Atg7 were capable of phagocytosing equivalent amounts of *A. fumigatus* conidia, but did not recruit LC3 to the phagosome. To determine if LAP was clinically significant, Beclin-1^flox/flox^, Rubicon-1**^−^**^/**−**^, and Atg7^flox/flox^ mice were challenged intranasally with *A. fumigatus*. A marked increase in IL-1β, IL-6, IL-12 and TNF-α was noted in LAP-deficient mice as compared to wild-type control. In addition, lung homogenate analysis revealed higher fungal colony forming units with delayed control of pathogen burdens critically linking LAP with control of *A. fumigatus* [13].

The association of LC3 and *A. fumigatus* was further extended by de Luca *et al.* to demonstrate a role for LC3-dependent inflammasome regulation. In CGD patients with NADPH oxidase mutations, de Luca *et al.* [12] explored the role of ROS production with autophagy as it relates to the control of *A. fumigatus*. The loss of ROS production was associated with higher levels of inflammasome/caspase activity as measured by IL-1β. In addition to enhanced caspase activity, a decrease in LC3 recruitment to *A. fumigatus* conidia was observed. In fact, p47phox^−/−^ mice, a model of CGD, reveal higher fungal burdens in a model of pulmonary aspergillosis establishing a link between NADPH oxidase/ROS production with LC3 and clearance of *Aspergillus* conidia [12]. Interestingly, use of anti-IL-1 receptor antagonist therapy (Anakinra) resulted in decreased *A. fumigatus* burden from the lung in a pulmonary model of invasive *Aspergillus*, suggesting that the dysregulation of caspase activity due to loss ROS by p47phox can be corrected [12]. de Luca *et al.* also demonstrate that in CGD patients with related colitis–an inflammatory bowel disease that has been related to dysregulation of autophagy and ROS production–is ameliorated following blockade of IL-1. In addition, the authors demonstrate restoration of LC3 localization to *A. fumigatus* conidia in monocytes from CGD patients treated with IL-1 receptor antagonist therapy. These data strongly link loss of NADPH oxidase activity to defective autophagy through inflammasome over activity; moreover the data show that interruption of the IL-1 receptor not only restores LC3–and presumed autophagy activity–but reestablishes control of fungal burden. The role of autophagy *versus* LAP in this process remains is an area for future study [12,14].

## 7. Conclusions

In recent years, proteins originally considered exclusive to macroautophagy are actively being redefined in the context of the host immune response to pathogenic fungi. Elucidating the role of autophagic proteins in the host response is still in its early stages, but the field has started to uncover how these proteins may function to protect the host against the threat of infection. Autophagic proteins were traditionally considered proteins that form autophagosomes to degrade intracellular microbes and organelles, but we now understand that these proteins can also affect phagocytosis and eventual killing of pathogens. However, the role of autophagic proteins depends on several variables in the context of infection: host species (e.g., mouse or human), type of fungal organism (e.g., *C. neoformans* or *C. albicans*), and even the strain of fungal organism (e.g., *C. albicans* SC5314 or UC820). Although further effort is required to elucidate the molecular details to LAP, these advances are defining how autophagic proteins function in immune signaling pathways and pathogen control. Continued research in this area will uncover new ways in which autophagy, an ancient cellular degradation system, functions in host fungal immunity.

## Figures and Tables

**Table 1:** Role of autophagy proteins in fungal infections.

Fungi	Effects of Autophagy Proteins	Reference
*Candida albicans*	*Candida albicans* induces a shift of LC3-I to LC3-II in macrophages.	[7,10,75]
	LC3 is recruited to phagosomes in macrophages infected with *Candida albicans.*	[7,10,15]
	LC3-II recruitment to phagosomes requires Dectin-1 –dependent Syk phosphorylation.	[7,10]
	LC3-II recruitment to phagosomes is required for killing of *Candida albicans* by macrophages.	[10]
	Knockdown of Atg5 in macrophages *in vitro* results in reduced phagocytosis and killing of *Candida albicans.* The conditional knockout of Atg5 in murine myeloid cells increases mortality in response to *Candida albicans in vivo*.	[15]
	Atg7 is essential in mouse myeloid cells for host resistance to *Candida albicans* by enhancing neutrophil recruitment *in vivo*.	[6]
	Conditional knockout of Atg7 in mouse myeloid-cells did not lead to increased mortality due to *Candida albicans.*	[9]
*Aspergillus fumigatus*	LC3-II is recruited to phagosomes containing *Aspergillus fumigatus.*	[11,13]
	LC3-II recruitment is Dectin-1 dependent and regulated by Syk kinase dependent ROS production.	[11]
	Knockdown of Atg5 in human macrophages results in a reduction in the number of fungal spores contained within acidified lysosomes and reduced fungal killing.	[11]
	Macrophages deficient in autophagy proteins (Beclin-1, Rubicon, Atg7, and NOX2) fail to recruit LC3-II to pathogen containing phagosomes and display defects in pathogen clearance.	[13]
	Human monocytes from CGD patients (defective ROS production) and CGD murine macrophages have minimal LC3 recruitment and increased release of IL-1β in response to *Aspergillus fumigatus*. Inhibition of IL-1β in CGD mice increases LC3 recruitment and autophagy gene expression, and protects CGD mice from invasive aspergillosis.	[12]

## Abbreviations

The following abbreviations are used in this manuscript:

LC3	Microtubule-associated light chain 3
LAP	LC3-associated phagocytosis
Syk	Spleen tyrosine kinase
Atg	Autophagy-related gene
APC	Antigen presenting cell
DC	dendritic cell
PAMP	Pathogen associated molecular pattern
CLR	C-type lectin receptor
NADPH	Nicotinamide adenine dinucleotide phosphate
NF-κB	Nuclear factor kappa-light-chain-enhancer of activated B cells
NFAT	Nuclear factor of activated T cells
NLR	Nod-like receptor
TLR	Toll-like receptor
PRR	Pattern recognition receptor
ITAM	Immunoreceptor tyrosine-activation motif
ULK1	Unc-51-like kinase1
ER	Endoplasmic reticulum
ROS	Reactive oxygen species
PI3K	Phosphatidylinositol-3-OH kinase
Rubicon	RUN domain protein as Beclin-1 interacting and cysteine-rich containing
mTOR	Mammalian target of rapamycin
MHC	Major histocompatibility complex
SNP	Single nucleotide polymorphism
CGD	Chronic granulomatous disease
